# Conditional Controlled Light/Dark Cycle Influences Exercise-Induced Benefits in a Rat Model with Osteoarthritis: In Vitro and In Vivo Study

**DOI:** 10.3390/jcm8111855

**Published:** 2019-11-02

**Authors:** Yunkyung Hong, Seunghoon Lee, Jeonghyun Choi, Yunho Jin, Jinyoung Won, Youngjin Jo, Yonggeun Hong

**Affiliations:** 1 Department of Physical Therapy, College of Healthcare Medical Science & Engineering, Inje University, Gimhae 50834, Korea; dangmoo777@naver.com (Y.H.); stormyboy@nate.com (S.L.); 2 Biohealth Products Research Center (BPRC), Inje University, Gimhae 50834, Korea; yiopiop0011@nate.com (J.C.); jynh33@naver.com (Y.J.); wonjy@kribb.re.kr (J.W.); defcon0815@hanmail.net (Y.J.); 3 Ubiquitous Healthcare & Anti-aging Research Center (u-HARC), Inje University, Gimhae 50834, Korea; 4 Department of Rehabilitation Science, Graduate School of Inje University, Gimhae 50834, Korea; 5 Department of Physical Therapy, Graduate School of Public Health, Inje University, Busan 47392, Korea; 6 Department of Medicine, Division of Hematology/Oncology, Harvard Medical School-Beth Israel Deaconess Medical Center, Boston, MA 02215, USA

**Keywords:** musculoskeletal homeostasis, physical exercise, environmental lighting, inflammation

## Abstract

Physical exercise has long been recommended as a treatment for osteoarthritis (OA), though its effects vary based on the exercise protocol. Here, we examined whether environmental lighting conditions influence the anti-inflammatory benefits of exercise in a rat model of OA. Moderate-intensity treadmill exercise (Ex) was performed for six weeks under a 12:12 h light/dark (L/D) cycle, and compared against rats housed in a 24 h continuous light (L/L) environment. L/L conditions were associated with serological changes shortly after OA induction, which exacerbated the inflammatory microenvironment in the joint. Differentiation capacity was also impaired in bone precursor cells isolated from normal rats maintained under L/L conditions, despite elevated inflammatory responses. Exercise training under L/L conditions led to increased corticosterone levels in the blood, which exacerbated the progression of cartilaginous and synovial lesions. Osteoporotic phenomena were also observed in exercise-trained rats maintained under L/L conditions, along with inflammation-induced catabolism in the gastrocnemius muscle. Aberrant light/dark cycle conditions were also found to be associated with suppression of splenic *Cry1* expression in exercise-trained rats, leading to dysregulation of immune responses. Taken together, these data suggest that lighting condition may be an important environmental factor influencing the exercise-induced benefits on OA.

## 1. Introduction

Osteoarthritis (OA) is the most common form of arthritis in older people, leading to functional disability due to pain [1]. Regular exercise has long been regarded as an effective method for alleviating pain in musculoskeletal diseases [2,3]. Physical exercise may provide benefits by disrupting chronic inflammatory pathways [4], though the extent of these exercise-induced benefits vary, depending on activity type and intensity. Low-to-moderate intensity aerobic exercise has been shown to limit OA-associated pain [5], and multimodal exercise therapy appears more effective in symptomatic control [6,7]; however, these vigorous activities are not recommended for patients with osteoarthritis [8]. A better understanding of the effects of workout intensity on disease outcomes is critical for creating effective exercise programs, although exercise intervention is underutilized, due to a fear of disease exacerbation [9].

As with internal factors, the external environment can significantly influence physiological responses to exercise. For example, it has been shown that thermoregulation, such as precooling prior to exercise, can improve performance in endurance activities [10]. Light/dark cycles have also been shown to impact motor activity in rodents [11], with aberrant light exposure proving detrimental to many biological systems, due to a disruption of diurnal hormone variations [12,13,14]. Melatonin, which is mainly secreted from the pineal gland during the night, is representative of the circadian hormones. In contrast, glucocorticoids are released from the adrenal gland, and are well established as a clock-based stress hormone [15]. Since these two hormones regulate peripheral physiology, by adjusting the internal clock in response to cyclic fluctuations in the environment, their levels should be maintained rhythmically in the body. Exercise training has been shown to differentially regulate the levels of these hormones, thereby restoring homeostasis; however, the molecular mechanisms underlying these exercise-induced benefits remain unclear. Here, we sought to determine the effects of environmental lighting conditions on interventional exercise in osteoarthritic rats.

## 2. Materials and Methods

### 2.1. Induction of Osteoarthritis and Exercise Training

Experimental procedures were approved by the Ethics Committee for Animal Care and Use at Inje University (approval no. 2012-030, 2017-021). Male Sprague-Dawley rats (8-week-old) weighing in the range 250–285 g were used as a model of osteoarthritis (OA). Unilateral knee osteoarthritis was induced by injecting joints with collagenase purified from the bacterium Clostridium histolyticum (Sigma-Aldrich, St. Louis, MO, USA) [16]. Rats were housed under differential light/dark conditions as follows: 12:12 light/dark (L/D); 24:00 h constant light (L/L) maintained under controlled temperatures. The rats were pre-adapted for 2 weeks, under conditions of L/D and L/L light/dark cycles, before collagenase injection. All rats were provided food and water ad libitum. OA rats were divided into three groups (*n* = 8 for each condition): OA sedentary (OS), OA exercise-trained under L/D conditions (OEx+L/D), and OA exercise-trained under L/L conditions (OEx+LL). A motor-driven treadmill was used for the moderate-intensity exercise. Pre-training (11 m/min, 20 min/day, 5 days/week) was initiated 1 week before OA induction. Exercise training was initiated at the same circadian time, regardless of lighting condition. The body weight of the animals was measured once per week for the duration of the study. After sacrifice, we excised the specimens at ZT14 (ZT0, 07:00) and measured the value of anteroposterior thickness of the osteoarthritic knee, as well as the wet weight of skeletal muscles, using an electronic balance.

### 2.2. Behavioral Assessments

Mechanical sensitivity was assessed by stimulating the hind paws via application of calibrated Von Frey filaments (North Coast Medical, Morgan Hill, CA, USA). The rats were placed on top of a wire mesh floor and acclimatized to the surroundings for at least 15 min. We began the testing using an “ascending stimulus” method, after verifying cessation of exploratory behavior [17]. The tip of the monofilament was then applied to the midplantar surface of the hind foot until the Von Frey hair began to bend, after which it was held in place for 5 sec. Stimulations were repeated at least 10 times, with sufficient intervals between applications, and the number of paw withdrawal responses was recorded. A filament, with 40% withdrawal response rates over 10 applications, was set as the baseline mechanical threshold for each animal. Additionally, the values of right-left linear distances were measured for analysis of functional locomotion, as previously described [18].

### 2.3. Enzyme-Linked Immunosorbent Assay (ELISA)

Serum was stored at −80 °C until analysis, then thawed. Measurements were conducted in duplicate using commercial ELISA kits: melatonin (Cloud-Clone Corp., Houston, TX, USA); corticosterone, TNFα, IgM (Abcam, Cambridge, MA, USA); IgG (Abnova, Neihu District, Taipei City, Taiwan). Immunoassay results were read with a fluorescence multi-detection reader (Bio-Tek Instruments, Winooski, VT, USA) at the indicated wavelength. Assay concentrations were quantitated using GraphPad PRISM software (GraphPad Software, La Jolla, CA, USA). A nonlinear regression analysis was used to derive an equation to predict the concentration in unknown samples.

### 2.4. Histomorphological Assessments

Hematoxylin and eosin staining was performed on decalcified specimens and analyzed using an Olympus DP70 microscope, using a 20× objective and digital camera (Olympus, Tokyo, Japan), connected to a computer. Ex vivo, micro-computed tomography (micro-CT) analysis was used to compare OA-induced changes in bone structure. Briefly, we analyzed the reconstructed images using CTAN software to obtain quantitative values on the bone structure after scanning the limbs. The trabecular bone, with thickness of 2 mm and 2 mm from the growth plate, was used as the region of interest for analysis.

### 2.5. Primary Cell Isolation and Culture

Primary osteoblasts (OB) were obtained by enzymatic digestion from the calvaria of adult Sprague-Dawley rats [19]. Briefly, calvarial bones were dissected from the head, cleaned of adhering soft tissues, and washed with phosphate-buffered saline (PBS). Calvarias were then cut into ~1-mm^3^ pieces and digested in enzymatic solution (2 mg/mL collagenase II in MEMα) twice at 37 °C for 30 min, with gentle shaking. Pieces were then further digested in a solution containing 0.25% trypsin and 0.1% EDTA for 30 min. Finally, the pieces were incubated in 2 mg/mL collagenase II solution. After digestion, the calvarial bone chips were suspended in a complete culture medium (10% FBS, 1% penicillin/streptomycin, 2 mM L-glutamine in MEMα). Primary osteoblasts, outgrown from the bone fragments, were then trypsinized, transferred into a new dish, and cultured at 37 °C in 5% CO_2,_ with humidification. When cells reached confluence (T0), osteogenic medium (50 µg/mL ascorbate, 10 nM dexamethasone, and 10 mM β-glycerophosphate in complete culture medium) was added for in vitro differentiation [20], with differentiation medium changed every 2 days. Bone marrow macrophages (BMMs) of Sprague-Dawley rats were isolated, as previously described [21]. Briefly, the ends of the tibial and femoral bones were cut off with scissors, and bone marrow cells were then flushed with washing medium (2% FBS, 1% penicillin/streptomycin in MEMα). After removing erythrocytes with hypotonic buffer, cells were cultured in complete culture medium (10% FBS, 1% penicillin/streptomycin in MEMα) for 24 h. Non-adherent cells were collected, seeded onto 24-well plates (5 × 10^5^ cells per well; T0), and cultured in osteoclastogenic medium (60 ng/mL M-CSF in complete culture medium). After a 2-day culture, 20 ng/mL RANKL was further added to the osteoclastogenic medium. Cells were then cultured for 6 days, with the differentiation medium changed every other day. Phase contrast microscopy was used to observe cell morphology.

### 2.6. TRAP Staining

Osteoclast differentiation was assessed by tartrate-resistant acid phosphatase (TRAP) staining. After 6 days of culture, cells were stained using a commercial TRAP kit (COSMO BIO, Tokyo, Japan), according to the manufacturer’s protocol. Cells with three or more nuclei were counted as multinucleated mature osteoclasts.

### 2.7. Quantitative Real-Time PCR Analysis

Total RNA was extracted using TRI-reagent (Sigma-Aldrich, St. Louis, MO, USA) and quantified using a Nanodrop (DeNovix, Wilmington, DE, USA). cDNA synthesis was performed using 1 µg of total RNA with random hexamers (Elpisbio, Daejeon, Korea). Real-time PCR was carried out using a Light Cycler 1.5 (Roche Instrument AG, Rotkreuz, Switzerland) with LightCycler FastStart DNA Master SYBR Green I (Roche, Rotkreuz, Switzerland). cDNA samples were amplified using specific primers (Table 1). Cycle temperatures and times were determined based on the manufacturer’s protocol. Each sample was assessed at least in duplicate. Average ΔCt values were calculated by subtracting the Ct values of the reference genes (*Actb*, *Gapdh*) from those of the target genes. The relative gene expression in the experimental group versus the control group was calculated using the 2−ΔΔCt method.

### 2.8. Western Blotting

Specimens were lysed in a protein extraction solution (Elpisbio, Daejeon, Korea), supplemented with a protease inhibitor cocktail (Roche, Rotkreuz, Switzerland). Concentrations of proteins were measured with the Bradford assay (Bio-Rad Laboratories, Richmond, CA, USA) using a Nanodrop (DeNovix, Wilmington, DE, USA). A total of 20 µg of protein per sample was separated by SDS-PAGE and transferred to a PVDF membrane (MerckMillipore, Billerica, MA, USA). The following primary antibodies were incubated overnight: rabbit polyclonal anti-ERK1/2, goat polyclonal anti-phospho ERK1/2, rabbit polyclonal anti-OPG, goat polyclonal anti-RANKL, rabbit polyclonal anti-COL2A1, rabbit polyclonal anti-MMP-13, goat polyclonal anti-TLR4, mouse monoclonal anti-MAFbx, mouse monoclonal anti-actin, mouse monoclonal anti-β-actin, rabbit polyclonal anti-GAPDH (Santa Cruz Biotechnology, Santa Cruz, CA, USA), rabbit polyclonal anti-AKT, rabbit polyclonal anti-phospho AKT (Cell Signaling Technology, Danvers, MA, USA), and rabbit polyclonal anti-iNOS (Proteintech, Rosemont, IL, USA). Chemiluminescent reagent (Pierce Biotechnology, Rockford, IL, USA) was used for visualization of band signals. Specific protein bands were quantified using the ImageJ software (NIH, Bethesda, MD, USA).

### 2.9. Statistical Analysis

Data are presented as the mean ± standard deviation (SD). Statistical significance was assessed using one-way analysis of variance (ANOVA) with the post hoc Tukey’s test, and also using student’s *t*-test. Differences were deemed statistically significant at a *p*-value < 0.05. All analyses were performed using the statistical software SPSS Network 24 (IBM, New York, NY, USA).

## 3. Results

### 3.1. Continuous Lighting Induces an Inflammatory Microenvironment in Osteoarthritic Joints

**Figure 1:**
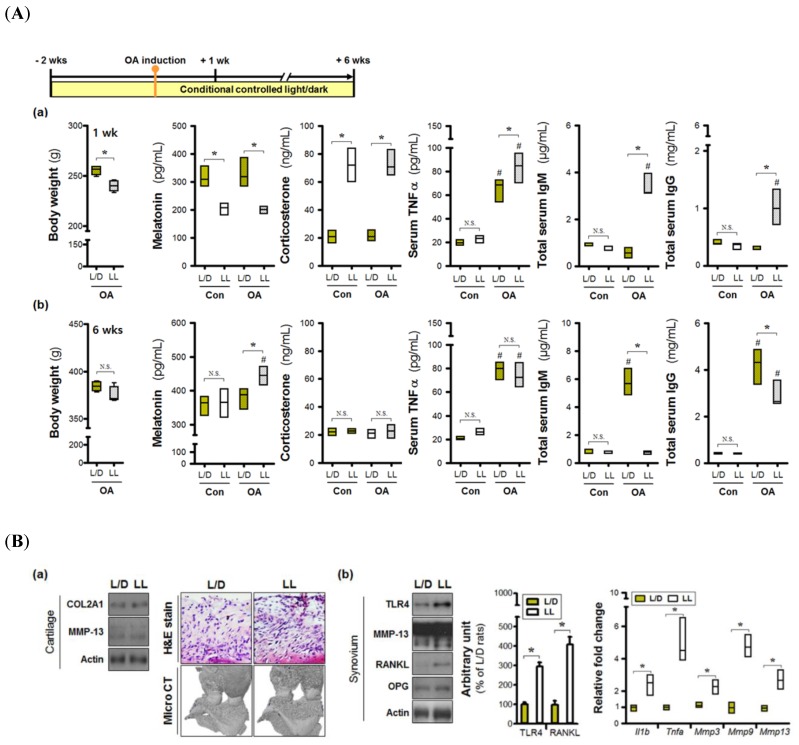
Constant-light-induced hormonal changes exacerbate synovial inflammation in the osteoarthritic joint. Different light/dark cycles were applied to osteoarthritic rats for 6 weeks. (**A**) Differences in hormones levels (melatonin, corticosterone), immunoglobulins (IgM, IgG), and TNFα in serum, as well as overall body weight, were observed at (**a**) 1 week and (**b**) 6 weeks after osteoarthritic induction. (**B**) Differences in disease outcomes associated with lighting conditions were limited to (**a**) cartilage destruction and were not observed in (**b**) synovial inflammation. Gene expression levels are shown relative to the L/D group. * *p* < 0.05 L/D vs. LL; # *p* < 0.05 Con vs. OA. N.S. not significant.

We determined the effects of environmental lighting conditions on joint inflammation in vivo. At 1-week post-OA induction, the average body weight of the L/L group was lower than that of the L/D group (Figure 1(Aa)), with clear intergroup differences in the levels of serum factors. Serum melatonin levels were lower in the L/L group, combined with higher concentrations of corticosterone. These phenomena represent the early physiological load induced by environmental light stress; however, arthritis-induced hormonal changes were not observed. TNFα levels were increased after OA induction, with higher levels seen under the 24 h lighting condition. Abnormal lighting also led to increases of serum immunoglobulins IgM and IgG, indicating early disruptions in serum homeostasis. Changes in serum immunoglobulin levels were also observed in the L/D group; however, these changes did not arise until 6 weeks post-OA induction (Figure 1(Ab)). Immunoglobulin levels were much higher in the L/D group at 6 weeks, while TNFα concentrations were similar between the groups. The continuous lighting environment also induced significant increases in melatonin levels at night, though these changes were limited to OA rats.

Next, we analyzed the damage in OA joint tissues. Histological changes in cartilage specimens, as well as levels of COL2A1 and MMP-13 proteins, were not different between groups, indicating that the degree of cartilage destruction may be similar (Figure 1(Ba)). However, there was evidence of more severe subchondral bone erosion as a result of micro CT in the L/L group. Although a difference in MMP-13 protein levels (both pro- and activated form) was not observed in the synovium, both TLR4 and RANKL proteins were elevated in the L/L group (Figure 1(Bb)). Furthermore, osteoarthritic synovial tissues expressed higher levels of pro-inflammatory (*Il1b*, *Tnfa*) and catabolic genes *(Mmp3*, *Mmp9*, *Mmp13*) in the L/D group, relative to the LL group. Together, these results suggest that continuous light exposure may promote the development of an inflammatory microenvironment.

### 3.2. Continuous Light Exposure is Injurious to Bone Cells

**Figure 2:**
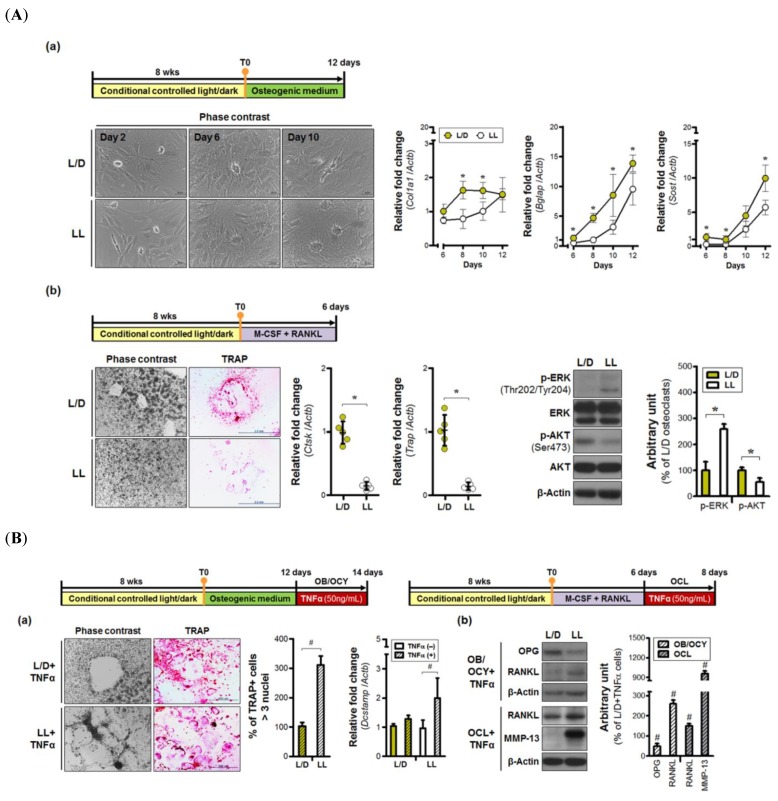
Detrimental effects of continuous lighting on bone cell biology. (**A**) Continuous lighting inhibits differentiation of precursor cells in mature (**a**) osteoblasts and (**b**) osteoclasts. (**a**) The relative fold changes were calculated relative to values from 6 days of calvarial osteoblasts isolated from the light/dark (L/D) group. (**b**) Levels of gene expression were compared relative to that of the L/D group. (**B**) Continuous light exposure increases not only cell fusion-mediated formation of multinuclear osteoclasts, but also the level of catabolic factors in bone cells in response to an inflammatory stimulus. The relative gene expression was measured by comparing values from each lighting condition to those from the TNFα-free group. T0, primary cell isolation; OB, osteoblast; OCL, osteoclast; OCY, osteocyte. OB magnification ×40; OC magnification ×40. * *p* < 0.05 L/D vs. LL; # *p* < 0.05 L/D+TNFα vs. LL+TNFα.

Calvarial osteoblasts were used to assess whether environmental lighting conditions influence osteogenic differentiation capacity. The ovoid type of osteoblasts was observed in both groups two days after the addition of the osteogenic medium (Figure 2(Aa)). Not only were osteoblasts evident, but early osteocyte morphology was also seen at 6 days. At 8 days of differentiation, maximum *Col1a1* expression was observed in cells isolated from L/D rats. In contrast, cells derived from the L/L group expressed lower amounts of *Col1a1* mRNA up to 10 days post-osteogenic induction. The original cell volume was significantly reduced in the L/D group at 10 days post-osteogenic induction, with long extending projections in direct contact with neighboring osteocytes. Similar levels of dendritic elongation and connection were not observed in the L/L group at the same time period. Levels of *Bglap* and *Sost* expression were also decreased in the L/L group, relative to the L/D group, indicating that continuous lighting conditions may delay osteogenic maturation.

Next, we examined the effects of continuous lighting on bone marrow-derived osteoclast differentiation in vitro. Multinucleated osteoclasts arose in response to M-CSF and RANKL treatment in the L/D group, but not in the LL group (Figure 2(Ab)). Furthermore, the number of TRAP-positive cells was decreased in the L/L group. Substantial decreases in the expression of *Ctsk* and *Trap* from osteoclasts were observed in the L/L group. Differences in activated intracellular signaling were also observed between groups, with significant phosphorylation of Akt in the cells from the L/D group, whereas the continuous lighting group showed activation of ERK signaling. Thus, abnormal light exposure might impair the generation of osteoclasts other than osteoblasts and osteocytes.

Next, TNFα was applied to various populations of osteoblasts (OB), osteocytes (OCY), and osteoclasts (OCL). Inflammatory stimulation induced osteoclast enlargement, compared to the original cell size, shown in Figure 2(Ab), with levels of TRAP+ multinucleated osteoclasts ~three-fold greater in the LL+TNFα group, compared to that of the L/D+TNFα group (Figure 2(Ba)). Expression of *Dcstamp* (a marker of osteoclast fusion) was also enhanced in cells isolated from continuously light-exposed rats, indicating that continuous light exposure resulted in greater cell-to-cell fusion in response to an inflammatory stimulus. Finally, continuous light exposure led to increases in both RANKL and MMP-13 protein levels in mature osteoclasts treated with TNFα (50 ng/mL). Osteoblasts and osteocytes exposed to continuous light induced not only down-regulation of OPG, but also up-regulation of RANKL, in response to TNFα treatment. These results mean that aberrant lighting conditions may enhance inflammatory-mediated activation of catabolic machinery.

### 3.3. Aberrant Lighting Conditions Causes a Reduction in Exercise-Induced Benefits in Inflamed Joints

**Figure 3:**
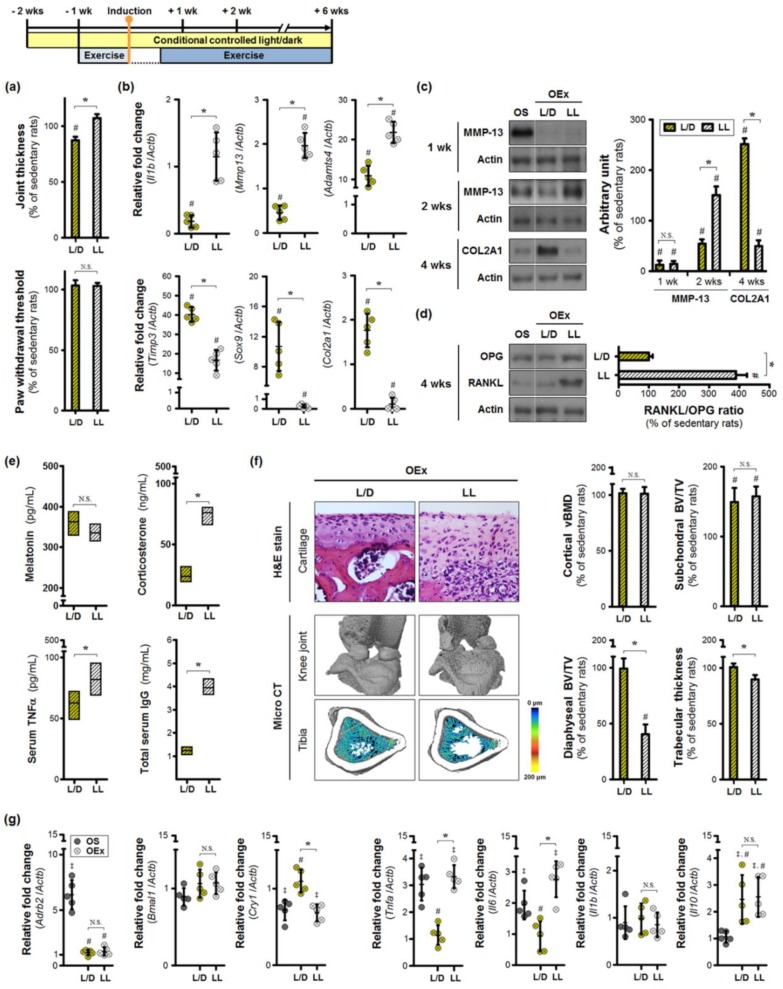
Moderate-intensity exercise training under continuous lighting environment amplifies catabolic signals during the progression of osteoarthritis. Different light/dark cycles were applied to osteoarthritic rats for 6 weeks. Additional treadmill exercise under each lighting condition was performed after the recovery period, following osteoarthritis induction. (**a**) Comparison of the values of joint thickness and withdrawal threshold are shown, along with alterations in (**b**) serum hormones (melatonin, corticosterone), pro-inflammatory cytokines (TNFα), and immunoglobulin (IgG). (**c**~**d**) Lighting conditions regulate exercise-induced changes in extracellular matrix turnover in cartilage specimens. The gene expression levels were examined relative to those seen in the sedentary group. (**e**) Synovial RANKL/OPG ratios were compared between lighting conditions. (**f**) Histological and micro-CT analyses are shown. vBMD, volumetric bone mineral density; BV/TV, bone volume fraction. Magnification ×40, scale bar = 100 μm. * *p* < 0.05 L/D vs. LL; # *p* < 0.05 Sedentary vs. Exercise-trained; N.S. not significant. (**g**) Exercise training under abnormal light/dark cycles disrupts splenic Cry1-mediated immune regulation. The relative gene expression levels were measured by real-time qPCR and compared with the control group. * *p* < 0.05 L/D vs. LL; ^‡^ *p* < 0.05 Con vs. OA; # *p* < 0.05 Sedentary vs. Exercise-trained; N.S. not significant.

Next, we examined the impact of moderate-intensity exercise in the context of different light/dark cycle conditions. Exercise training under continuous lighting conditions induced greater thickening of the knee joint at 6 weeks post-osteoarthritis induction (Figure 3a). However, the mechanical threshold of the paw was not altered. Exercise training attenuated changes in serum melatonin resulting from continuous light exposure, but also led to higher levels of corticosterone, TNFα, and IgG (Figure 3b). Expression of inflammatory (*Il1b*) and catabolic (*Mmp13*, *Adamts4*) genes were upregulated in the cartilage of exercise-trained rats (Figure 3c). Consistent lighting lowered the expression of *Timp3*, *Sox9*, and *Col2a1* in the OEx+LL group. Compared to the sedentary OA rats, the exercise-trained rats exhibited lower levels of activated MMP-13 at 1 week (Figure 3d). However, after 2 weeks, prolonged exercise under aberrant light conditions led to a greater activation of MMP-13 than that seen in sedentary rats. COL2A1 protein levels were lower in the OEx+LL group at 4 weeks, in sharp contrast to exercise-trained rats housed under normal light/dark conditions. This shows that the highest reduction of COL2A1 protein expression occurred in the OEx+LL group at 4 weeks. Consequently, the exercise-trained rats under normal light/dark cycle had the highest abundance of COL2A1 protein. The inflammatory cell infiltrates were visible within the cartilaginous tissue (Figure 3f). These results suggest that cartilage destruction may be exacerbated by exercise training under continuous lighting conditions. Continuous lighting increased the RANKL:OPG ratio in the synovium of the inflamed joint, due to enhanced expression of RANKL (Figure 3e), indicating a high probability of osteoclast activation. The reconstructed micro-CT results exhibited severe trabecular bone loss in the tibial shaft of exercise-trained rats housed under continuous lighting conditions (Figure 3f). Reductions in bone volume fraction (BV/TV) were accompanied by similar decreases in mean trabecular thickness. Exercise-training increased the subchondral bone volume fraction independently of lighting conditions, with no alterations in the microarchitecture of cortical bone.

Finally, we investigated the possibility of splenic alterations due to continuous light exposure. Splenic expression of *Adrb2*, indicative of greater activation of the sympathetic nerves, was significantly upregulated in sedentary rats with joint inflammation (Figure 3g). Moderate exercise training reduced the levels of *Adrb2* to levels similar to those of control rats; these effects occurred independent of lighting conditions. Expression of the core clock gene *Bmal1* was not altered in the spleens of OA rats, while *Cry1* mRNA abundance was decreased. Concurrently, there was an upregulation of splenic *Tnfa* and *Il6* expression; differences in *Il1b* expression levels were not statistically significant. Exercise training restored the expression of *Cry1*, *Tnfa*, and *Il6* to baseline levels in rats housed under normal light/dark conditions, but not in those exposed to continuous light. Interestingly, anti-inflammatory *Il10* expression was increased in response to exercise training regardless of lighting environment. These results suggest that regular exercise may serve as a way to regulate systemic inflammation, by resetting an impaired splenic clock.

### 3.4. Exercise Training under an Abnormal Light/Dark Cycle Facilitates Muscular Inflammation

**Figure 4:**
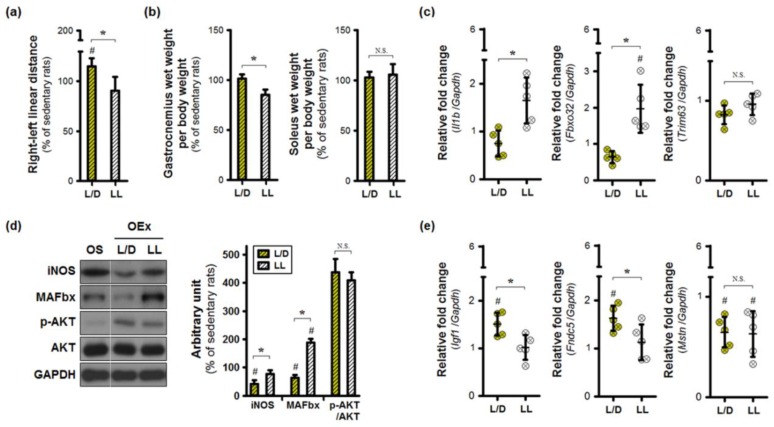
Exercise training under aberrant lighting conditions contributes to muscular inflammation. (**a**) Right-to-left linear distances during locomotion (n = 32 for each condition) were normalized, relative to those of sedentary rats. (**b**) Wet muscle weight was measured, not only in the gastrocnemius, but also in the soleus. (**c**) Expression of inflammatory (Il1b) and catabolic markers (Fbxo32, Trim63) was measured by real-time qPCR in the gastrocnemius muscle. (**d**) The proteins involved in skeletal muscle metabolism were analyzed by immunoblotting in the gastrocnemius. (**e**) Expression of *Igf1*, *Fndc5*, and *Mstn* mRNA were analyzed by real-time qPCR and compared against values from the sedentary group. * *p* < 0.05 L/D vs. LL; # *p* < 0.05 Sedentary vs. Exercise-trained; N.S. not significant.

Under the standard 12:12 h light/dark cycle, exercise training improved the right-to-left distances, indicating the capacity to support body weight with the arthritic limb during locomotion (Figure 4a). In contrast, the continuous light environment limited exercise-induced increases in linear distance. Such a functional change may be caused by inappropriate remodeling of the skeletal muscle. To test this hypothesis, we analyzed the gastrocnemius, as well as the soleus, in osteoarthritic limbs. The wet weight of the gastrocnemius alone was lower in the OEx+LL group, compared to the OEx+L/D group (Figure 4b). Continuous lighting increased the expression of *Il1b* and *Fbxo32* mRNA in the gastrocnemius of exercise-trained rats, which was found to be higher than levels in sedentary rats (Figure 4c). MAFbx proteins encoded by *Fbxo32* were more abundant in the OEx+LL group (Figure 4d). Exercise-trained rats housed under standard L/D conditions exhibited reduced levels of MAFbx as well as iNOS proteins, although AKT phosphorylation levels were enhanced in both training groups. Next, we determined the interaction between skeletal muscle and bone, to identify the conditions necessary to maintain musculoskeletal homeostasis (Figure 4e). Exercise training under normal light/dark conditions elevated the levels of *Igf1* and *Fndc5* expression; these effects were not observed in the OEx+LL group. Regulation of *Mstn* expression was affected by the presence of exercise training alone. These results suggest that the repeated contraction of skeletal muscle under normal light/dark conditions may be capable of inhibiting osteoporosis in the tibial shaft.

## 4. Discussion

Our findings demonstrate the importance of lighting conditions for optimizing exercise-induced health benefits. Aberrant lighting conditions can lead to alterations in normal biological rhythms, due to the importance of the daily light/dark cycle in generating optimal circadian rhythms in both humans and animals. Shortening of the light/dark cycle contributes to the disruption of clock gene expression in both central and peripheral organs, through abnormal control of systemic time cues [11,22]. Their regulation is also affected by the duration of light exposure throughout the day [23]. Here, we applied continuous lighting to induce arrhythmicity, and measured the levels of melatonin in the sera. Circulating melatonin is one of the primary photic signal transmitters, with 2 weeks of continuous nighttime light exposure significantly dampening nocturnal concentration in both humans and rodents [24,25].

Diurnal variations in melatonin secretion have also shown to be disrupted in response to lighting conditions [26]. In the present study, continuous lighting induced an early, rather than late, reduction in melatonin levels. Although we are unsure whether the daily rhythm of melatonin secretion was impaired, nighttime levels were accompanied by more severe inflammatory conditions in the constant-light environment. Because either biophysical or psychological stress can elevate melatonin as well as its metabolites [27,28], exacerbated joint inflammation may be considered a systemic stressor. High levels of circulating norepinephrine, released from the adrenal medulla during stress, stimulate pinealocytes via sympathetic innervation [28], which may be analogous to the explanation for the increase in melatonin described here. Inflammatory stress-induced activation of the sympathetic nervous system was observed at later time points in the arthritic spleen, leading to a greater production of immunoglobulins and pro-inflammatory cytokines. The pro-inflammatory actions of the sympathetic nervous system were previously observed in the early phases of autoimmune arthritis [29]. In our data, continuous lighting resulted in a shift in endogenous antibody production from the late phase to the early phase. Therefore, it is assumed that continuous light exposure may alter the characteristics of collagenase-induced osteoarthritis [30] to that of an early autoimmune-like phenotype.

It is interesting to note that elevated endogenous melatonin production may act as the etiologic agent of rheumatic arthritis [31]. Here, we observed features of synovial inflammation, in combination with elevated melatonin levels, in late stage OA rats. Exogenous melatonin treatment in inflammatory diseases has been shown to aggravate disease phenotypes via the attenuation of *Cry1* gene expression [32,33]. These observations are not surprising, given the role of the molecular clock system in regulating immune function [34]. The circadian clock protein CRY, inter alia, contributes to the production of pro-inflammatory cytokines such as TNFα, IL1β, and IL6 [35,36,37]. Consistent with this, we observed that a sedentary condition contributes to an increased expression of pro-inflammatory genes, such as *Tnfa* and *Il6,* in the spleen, in response to *Cry1* inhibition. These pro-inflammatory phenotypes are analogous to those seen during exercise training in the constant-light environment, even if the difference in melatonin was nonsignificant, due to the regulatory effect of exercise on circulating melatonin levels. Instead, the concentration of corticosterone was much higher in the exercise-trained rats under conditions of chronic light exposure.

Besides melatonin, secretion of glucocorticoids from the adrenal cortex can also be altered by environmental lighting stress [26,38,39,40]. These stress-induced increases in corticosterone secretion promote chronic, low-grade inflammation throughout the body [41], despite the powerful anti-inflammatory and immunosuppressive actions of these compounds [42]. Furthermore, the higher basal levels of plasma corticosterone alone have been shown to confer pro-inflammatory properties [43,44]. Conditions of chronic stress induced not only a reduction in glucocorticoid sensitivity, but also the development of resistance, thereby increasing vulnerability to exaggerated inflammatory responses [45]. In the current study, continuous exposure to light for 3 weeks was linked to higher nighttime serum corticosterone levels, leading to the rapid inflammatory responses associated with higher levels of circulating TNFα and immunoglobulins. These conditions were also observed after long-term exercise training in rats housed under continuous lighting conditions, suggesting that aberrant lighting conditions may reduce the potential health benefits of exercise.

Excessive level of circulating glucocorticoids is a risk factor for osteoporosis, hypertension, and metabolic syndrome [15]. Of these conditions, the underlying mechanism responsible for glucocorticoid-induced osteoporosis remains unclear. Even though early glucocorticoid-challenged rats became insensitive to chronic light exposure over time, its detrimental effects on the skeleton were persistent. Both osteoblasts and osteoclasts, isolated from the rats exposed to constant-light, exhibited clear patterns of delayed differentiation. Glucocorticoid signaling via monomeric receptors interrupts bone formation by directly inhibiting osteoblast differentiation [46]. Of note, it has been recently reported that glucocorticoids suppress the function of both osteoclasts and osteoblasts [47]. Furthermore, the osteolytic activity of osteoclasts is influenced by inflammatory signals (e.g., RANKL) [48]. The factors regulating bone biology can be secreted from extra-skeletal tissues adjacent to the joint, including the synovium, skeletal muscles, and ligaments [49]. Here, exposure to constant-lighting conditions alone was sufficient to induce an inflammatory response in arthritic animals, and was accompanied by an increase in RANKL protein.

As environmental lighting conditions change, changes in both the serum hormone concentrations, and in sleep-cycles, occur. The disruption of sleep-wake cycles, resulting from chronic environmental disturbance, may induce pathological alteration in the cartilage [50]. To reach a clearer conclusion, further study of the how pattern of sleep-wake cycles is associated with changes in cellular signaling of the cartilage is required.

**Figure 5:**
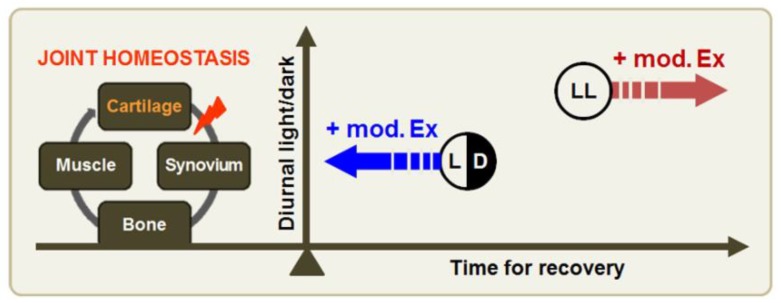
Exercise-induced benefits depend on environmental lighting conditions. Joint homeostasis is organically regulated by the actions of cartilage, synovium, bone, and skeletal muscles. Inflamed cartilage disrupts the homeostatic balance in the osteoarthritic joint; the degree to which this occurs might be aggravated under constant-lighting conditions. Additionally, joint damage may be exacerbated, instead of restored, when regular, moderate-intensity exercise is added in the context of aberrant light/dark cycles. Thus, lighting condition might be an important environmental factor when looking to restore homeostasis following joint damage.

Therefore, environmental conditions must be considered when developing an exercise intervention plan to treat arthritic patients, as aberrant light/dark cycles have been shown to accelerate joint inflammation, via disruption of circadian rhythms, co-regulated by melatonin and glucocorticoids (Figure 5).

## Figures and Tables

**Table 1:** Oligonucleotide primers used for PCR.

Gene	Primer Sequence (5′-3′)	Size (bp)	GenBank Accession No.
*Actb*	F: taa aga cct cta tgc caa cac agt R: cac gat gga ggg gcc gga ctc atc	241	NM_031144.2
*Gapdh*	F: ctc agt tgc tga gga gtc cc R: att cga gag aag gga ggg ct	120	NM_017008.4
*Adamts4*	F: agc ctt taa gca tcc aag ca R: gga ggg ttt agg cct ttc tg	153	NM_023959.1
*Adrb2*	F: aca cgg gaa tga cag cga ctt c R: cga tcc act gca atc acg cac	384	NM_012492.2
*Bglap*	F: tcc aag cag gag ggc agt aag R: taa acg gtg gtg cca tag atg c	194	NM_013414.1
*Bmal1*	F: gtc gaa tga ttg ccg agg aa R: ggg agg cgt act tgt gat gtt c	101	AB015203
*Col1a1*	F: tct gac tgg aag agc gga gag R: gag tgg gga aca cac agg tct	112	NM_053304.1
*Col2a1*	F: ggt ttg gag aga cca tga acg g R: gtc aac aat ggg aag gcg tga g	350	NM_012929.1
*Cry1*	F: gcc tca gtc cct tct aat cc R: tcc cgc atg ctt tcg tat c	284	NM_198750.2
*Ctsk*	F: ctg gga gac atg acc agc gaa g R: tgc act tag ctg cct ttg cc	433	NM_031560.2
*Dcstamp*	F: tgc aac cta agg gca aag agc R: gag gcc agt gct gac tag gat g	309	NM_029422.4
*Fbxo32*	F: gca aaa cat aag act cat acg R: gta gag tgg tct cca ttc g	134	NM_133521.1
*Fndc5*	F: ctc agc aga aga agg atg tga g R: cat ggt cac ctc atc ttt gtt c	221	NM_001270981.1
*Igf1*	F: cgc tct tca gtt cgt gtg tg R: cgg aag caa cac tca tcc ac	114	NM_001082477.2
*Il1* *b*	F: aac aaa aat gcc tcg tgc tg R: ttg tcg ttg ctt gtc tct cc	124	NM_031512.2
*Il6*	F: cac aga gga tac cac cca ca R: cac aaa ctc cag gta gaa acg g	277	NM_012589.2
*Il10*	F: taa ctg cac cca ctt ccc agt c R: cat tct tca cct gct cca ctg c	350	NM_012854.2
*Mmp3*	F: tga gag cag tgc aga act gtg g R: ctt gtg cat cag ctc cat agt g	296	NM_133523.3
*Mmp9*	F: gct atg gtt aca ctc ggg ca R: tgg cct tta gtg tct cgc tg	129	NM_031055.1
*Mmp13*	F: agg cct tca gaa aag cct tc R: gag ctg ctt gtc cag gtt tc	226	NM_133530.1
*Mstn*	F: gct ggc cca gtg gat cta aat g R: tga ttg ttt ccg tgg tag cgt g	304	NM_019151.1
*Sost*	F: gta cat gca gcc ttc gtt gct g R: act ggt tgt gga agc ggg tga g	490	NM_030584.1
*Sox9*	F: aca acg caa gct tct gca ag R: aca ctc tcc aac cac agc ag	111	NM_080403.1
*Timp3*	F: ggc caa agt ggt ggg aaa gaa g R: ccc acc tct cca caa agt tgc	245	NM_012886.2
*Tnfa*	F: cta ctg aac ttc ggg gtg atc R: ctt gtc cct tga aga gaa cct g	292	NM_012675.3
*Trap*	F: gat cac ctt ggc aat gtc tcg R: ggc tga caa agt cgt cgg aat	175	NM_019144.2
*Trim63*	F: agg tga agg agg aac tga g R: aac tgc tct cgg tac tgg	148	NM_080903.1
